# Developmental Eye Movement (DEM) Test Norms for Mandarin Chinese-Speaking Chinese Children

**DOI:** 10.1371/journal.pone.0148481

**Published:** 2016-02-16

**Authors:** Yachun Xie, Chunmei Shi, Meiling Tong, Min Zhang, Tingting Li, Yaqin Xu, Xirong Guo, Qin Hong, Xia Chi

**Affiliations:** 1 Department of Children Health Care, Nanjing Maternity and Child Health Care Hospital Affiliated with Nanjing Medical University, Nanjing, China; 2 Institute of Pediatrics, Nanjing Medical University, Nanjing, China; 3 Dachang Community Health Service Centers, Liuhe District, Nanjing, Jiangsu, China; Eberhard Karls University of Tuebingen Medical School, GERMANY

## Abstract

The Developmental Eye Movement (DEM) test is commonly used as a clinical visual-verbal ocular motor assessment tool to screen and diagnose reading problems at the onset. No established norm exists for using the DEM test with Mandarin Chinese-speaking Chinese children. This study aims to establish the normative values of the DEM test for the Mandarin Chinese-speaking population in China; it also aims to compare the values with three other published norms for English-, Spanish-, and Cantonese-speaking Chinese children. A random stratified sampling method was used to recruit children from eight kindergartens and eight primary schools in the main urban and suburban areas of Nanjing. A total of 1,425 Mandarin Chinese-speaking children aged 5 to 12 years took the DEM test in Mandarin Chinese. A digital recorder was used to record the process. All of the subjects completed a symptomatology survey, and their DEM scores were determined by a trained tester. The scores were computed using the formula in the DEM manual, except that the “vertical scores” were adjusted by taking the vertical errors into consideration. The results were compared with the three other published norms. In our subjects, a general decrease with age was observed for the four eye movement indexes: vertical score, adjusted horizontal score, ratio, and total error. For both the vertical and adjusted horizontal scores, the Mandarin Chinese-speaking children completed the tests much more quickly than the norms for English- and Spanish-speaking children. However, the same group completed the test slightly more slowly than the norms for Cantonese-speaking children. The differences in the means were significant (*P*<0.001) in all age groups. For several ages, the scores obtained in this study were significantly different from the reported scores of Cantonese-speaking Chinese children (*P*<0.005). Compared with English-speaking children, only the vertical score of the 6-year-old group, the vertical-horizontal time ratio of the 8-year-old group and the errors of 9-year-old group had no significant difference (*P*>0.05); compared with Spanish-speaking children, the scores were statistically significant (*P*<0.001) for the total error scores of the age groups, except the 6-, 9-, 10-, and 11-year-old age groups (*P*>0.05). DEM norms may be affected by differences in language, cultural, and educational systems among various ethnicities. The norms of the DEM test are proposed for use with Mandarin Chinese-speaking children in Nanjing and will be proposed for children throughout China.

## Introduction

Reading disability, like obesity, has become an important public health problem [1]. Children with poor reading skills include those with dyslexia, those with non-dyslexic reading disabilities, those with lower linguistic cognition and non-linguistic perceptual cognitive processing skills and those with other problems. Children with poor reading skills have reading comprehension difficulties and tend to dislike and avoid reading, resulting in a lack of reading experience that severely influences their ability to acquire knowledge compared with their peers [1, 2]. These children cannot establish a solid reading comprehension foundation, which affects their subsequent academic achievement, creating a vicious cycle. Therefore, the early identification of children at risk of developing reading problems is more economical and beneficial than later intervention and treatment.

Visual and auditory processing disorders result in poor reading skills; in particular, visual processing is a prerequisite for completing reading tasks [3]. Eye movements, including saccades, fixations and regressions, are the most important skills in reading [4, 5]. Children with developmental dyslexia (DD) exhibit abnormal eye movements, including an increased number of saccades, a long fixation or regression, and irregular regression distances [6]. The relationship between eye movement quality and reading difficulty has been well documented [7–10]. The morbidity of reading problems in individuals from countries where a phonetic language is spoken ranges from 5 to 10 percent [11–12]. However, to date, there has been no report of the morbidity of reading problems in China. Therefore, the evaluation of eye movements in children could help to identify reading problems at their onset and facilitate a preliminary evaluation that could provide a foundation for further research in the Chinese population.

The Developmental Eye Movement (DEM; Bernell Corp., Mishawaka, IN) test is one of the most common visual-verbal tests for evaluating ocular motor control and rapid automatized naming [13–15]. Since its introduction in 1987, this test has been widely used by optometrists [16]. In addition to allowing a thorough evaluation of eye movement functions, including fixation, saccade and regression, this test can evaluate visual information processing during reading. Because of its advantages, it has become a standardized clinical test and is highly recommended for the evaluation of children with reading problems, but not for dyslexic children [16]. However, to date, there is no established norm for the use of the DEM test with Mandarin Chinese-speaking Chinese children. Hence, in this study, we aimed to a) establish normative values for the DEM test for the Mandarin Chinese-speaking population in China and b) compare the obtained values with the published normative values for English- [16], Spanish- [17], and Cantonese-speaking Chinese children [18].

In this study, we aimed to develop normative values for the use of the DEM test to evaluate Mandarin Chinese-speaking children aged 5 to 12 years in Nanjing, China.

## Methods

### Participants

This study was approved by the ethics committee of the Nanjing Maternal and Child Health Hospital of Nanjing Medical University, where the investigation was conducted (2012 (12)). A total of 1,206 children aged 5–6 years old and 3,586 children aged 7–12 years old participated in this study. The random stratified sampling method was used to recruit children from eight kindergartens and eight primary schools in the main urban and suburban areas of Nanjing. The subjects were Chinese-speaking children aged 5 to 12 years. An age/grade-matching criterion was used for each subject. These children shared similar socio-cultural and educational backgrounds, and there was no bias caused by selection from clinical referral populations. Permission and informed consent were obtained from the principals and the children’s parents or guardians. The subjects were selected from first- to eighth-grade classrooms according to the following inclusion criteria: 5 to 12 years of age, regular classroom attendance, a near-point visual acuity of 1.0 (decimal scale) at 40 cm, and successful performance on the DEM pretest. The exclusion criteria included the following: a lack of adequate number-naming skills (for children older than 5 years) as determined by the DEM pretest, a neurological disease or physical disability, a diagnosis of mental retardation, a failure to complete the DEM test because of an inability to stay on task even after redirection, and great difficulty with the horizontal task (for example, keeping a finger in one place or becoming completely lost). We used the random-number technique and chose each mantissa of 2, 5 and 8 as our participants. The number of participants excluded from each group were 1, 3, 4, 6, 7, 9, and 0. These exclusions reduced the final sample size from 4,792 to 1,425 (n = 1,425; 697 males and 728 females). To the best of our knowledge, no systematic sample selection biases existed. Data were collected September to December 2012 and March to June 2013.

### DEM test

The DEM test comprised a pretest card and three 216×279-mm test cards from the DEM test for Cantonese-speaking children [18]. The test process was simple and proceeded as follows: a child was asked to read the numbers on the test cards aloud in the order in which they appeared, and the examiner recorded the time taken and the errors the child made. The pretest consisted of 10 single-digit numbers separated by equal spacing, and it was used to confirm that the child was able to name simple numbers without difficulty. The two subtest cards (Tests A and B) consisted of 80 numbers and were divided into two groups of 40 single-digit numbers. On each card, the 40 numbers were arranged into two vertical columns of 20 numbers each. Tests A and B served to determine the child’s automaticity for reading vertically aligned numbers. The third test card (Test C) consisted of the same 80 numbers arranged in a horizontal array of 16 rows with 5 numbers each. The first and fifth number of each row were aligned down the page; however, the second, third and fourth numbers in each row were randomly spaced.

### Procedures [18]

The standard DEM test was administered to each student by two experienced examiners in accordance with the test norms for Cantonese-speaking children [18] and other test instructions [17, 19]. The test was conducted in a quiet room in the school. For the pretest, the children were asked to read the 10 single-digit numbers out loud. If the child read all of the numbers correctly in 12 seconds or less, he/she passed the pretest, indicating that he/she could see and read the numbers clearly on the DEM test chart using his/her habitual visual abilities.

After the tests were completed, the following four scores were derived: the “vertical score” (the total time required to complete Tests A and B), the “horizontal score” (the time needed to complete Test C, with an adjustment according to the errors made), the “error score” (the total number of errors made in Test C), and the “ratio score” (the ratio of the horizontal score to the vertical score). Four types of reading errors were possible: substitution, omission, addition, and transposition. Only the “omission” and “addition” errors affected the actual reading time; therefore, the horizontal score was adjusted using the following formula (from the DEM manual): adjusted horizontal score = Test C (time in seconds)×80/(80-omission+addition) [18]. Finally, a vertical score, an adjusted horizontal score, an error score, and a ratio score were obtained for each child.

### Reliability and validity of the test

The time reliability test and tester reliability test were conducted separately using re-measurements to evaluate the reliability of our localized DEM test. To evaluate the time reliability of the test, we re-tested a random sample of 180 children (male:female = 1:1.1) approximately two weeks after the first (T1) test. To assess tester reliability, we switched the two testers; they re-tested a random sample of 180 children, and the test results were compared. For the internal consistency validity test, we analyzed the correlation coefficients among the three eye movement indices.

### Statistical analyses

The two researchers entered the data using EpiData 3.0 software (EpiData Association, Odense, Denmark). A uniqueness check, a double check, and a logic check were conducted to ensure that the data were completely correct. The data were then analyzed using SPSS 17.0 (SPSS, Chicago, IL). The DEM scores are shown as the mean±SD. Qualitative and count data are shown as percentages. In addition, the χ^2^ test was used to compare qualitative or count data. An independent *t* test was used to compare the DEM indices for the different languages (Mandarin vs Cantonese; Mandarin vs English; Mandarin vs Spanish) for different age groups. The Tukey-Kramer multiple comparison test was used as a post hoc test after ANOVA, and the *t* test was used for simple comparisons of data between two groups. A *P* value less than or equal to 0.05 was considered statistically significant.

## Results

### Study participant information

A total of 1,425 children (n = 1,425; 697 males and 728 females) participated in this study. The participants were recruited from eight kindergartens and eight primary schools in the main urban and suburban areas of Nanjing, China, using a random stratified sampling method. The children were distributed among eight age groups and ranged in age from 5 to 12 years. Two experienced pediatricians conducted the DEM test in Mandarin, thus preliminarily establishing the test norms for children in Nanjing. The participants’ male-to-female ratio was 1:1.04. All of the children completed the test, and their complete information was obtained. The demographic data of subjects in all age groups are shown in Table 1.

**Table 1:** Number of males and females in each age group.

	5 y~	6 y~	7 y~	8 y~	9 y~	10 y~	11 y~	12 y~	Total
Male	67	114	87	100	70	93	85	81	697
	48.9%	47.9.%	47.5%	51.3%	44.9%	50.5%	51.2%	48.8%	48.9%
female	70	124	96	95	86	91	81	85	728
	51.1%	52.1%	52.5%	48.7%	55.1%	49.5%	48.8%	51.2%	51.1%
Total	137	238	183	195	156	184	166	166	1425
	100.0%	100.0%	100.0%	100.0%	100.0%	100.0%	100.0%	100.0%	100.0%

### A comparison of the DEM scores for each gender

There were no significant differences in the eye movement index scores between genders (Vertical score: *t =* -0.671, *P*>0.05; Horizontal score: *t =* -0.825, *P*>0.05; Ratio score: *t =* 1.142, *P*>0.05; *t =* 0.946, *P*>0.05; Table 2).

**Table 2:** Comparison of the DEM scores according to gender.

		Male	Female	*t*	*P*
	n	697	728		
5~12 years old	Vertical	57.35±17.40	55.91±16.93	-0.671	0.390
	Horizontal	77.7±29.05	80.34± 29.89	-0.825	0.495
	Ratio	1.30±0.27	1.36±0.30	1.142	0.161
	Error	5.38±6.34	6.12 ±7.28	0.946	0.307

### The mean DEM scores for each age group

**Fig 1:**
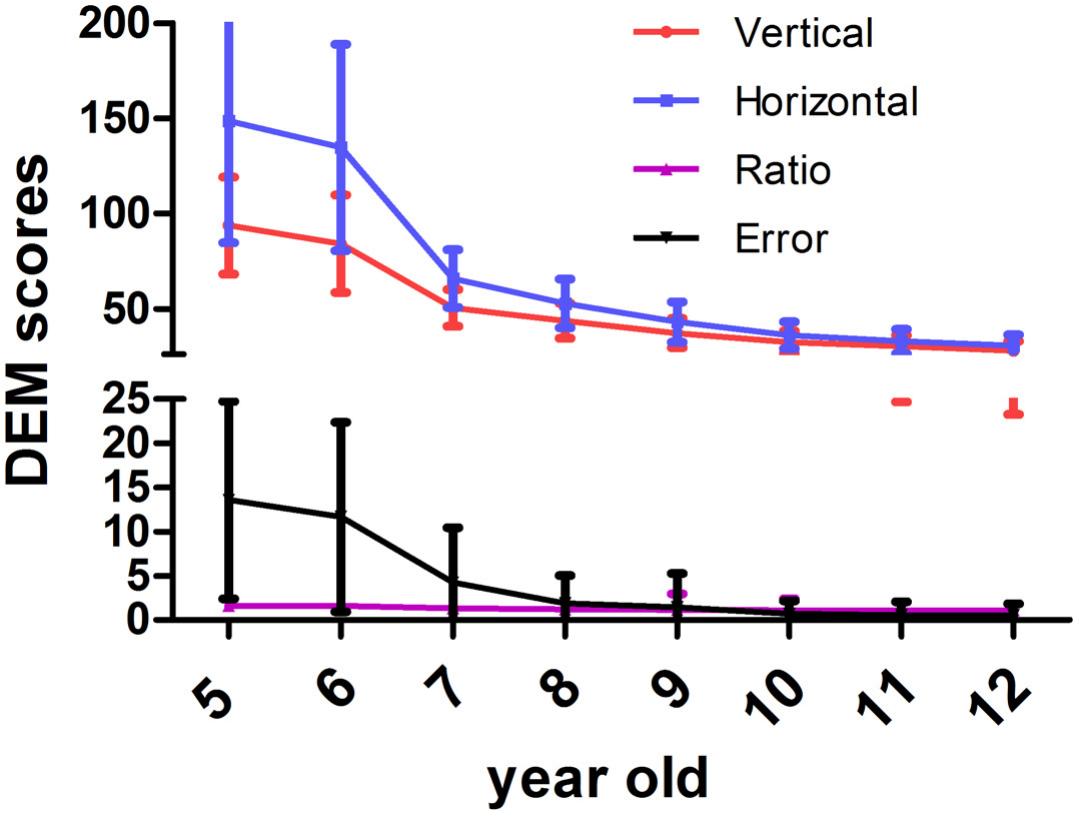
The mean DEM scores for each age group. The duration of each eye movement index decreased with age.

Based on the method and formulation described above, we calculated all of the DEM scores for each subject. The mean results are shown in Table 3. The DEM test showed a decrease with age in the four eye movement indices (vertical score, horizontal score, vertical-horizontal ratio and total errors), and significant differences were observed among the indices. This result suggests that the duration of each eye movement index decreased with age (Fig 1, Table 3). In comparison, the DEM scores for the horizontal score and vertical score were similar for the 10-, 11- and 12-year-old age groups (Fig 1, Table 3). The ratios and total errors did not significantly different among the 8-, 9-, 10-, 11- and 12-year-old age groups (Fig 1, Table 3). These results indicate that reading speed plateaus when children reach a specific age.

**Table 3:** DEM scores for Chinese (Mandarin)-speaking Chinese children by age group.

Age Group(years)	Vertical		Horizontal		Ratio		Error	
	$\bar{x}$±SD	95%CI	$\bar{x}$±SD	95%CI	$\bar{x}$±SD	95%CI	$\bar{x}$±SD	95%CI
5~	88.31±25.91	83.93~92.69	140.86±58.87	130.91~150.81	1.60±0.49	1.52~1.68	12.50±10.92	10.66~14.35
6~	62.84±17.97	60.55~65.14	86.67±34.10	82.32~91.03	1.38±0.28	1.34~1.41	7.17±8.22	6.12~8.22
7~	50.48±9.72	49.06~51.89	65.86±15.26	49.06~51.89	1.31±0.22	1.27~1.349	4.26±6.19	3.35~5.16
8~	43.64±9.14	42.35~44.93	52.76±12.74	50.96~54.56	1.21±.20	1.18~1.25	1.86±3.18	1.41~2.31
9~	37.18±7.76	35.9~38.41	43.03±10.66	41.34~44.71	1.16±.18	1.13~1.19	1.45±3.83	0.84~2.06
10~	32.33±6.04	31.45~33.20	36.04±6.98	35.03~37.06	1.12±.13	1.10~1.14	0.71±1.48	0.49~0.92
11~	30.35±5.67	29.48~31.22	32.83±6.48	31.84~33.82	1.09±.14	1.06~1.11	0.55±1.51	0.31~0.78
12~	28.06±4.80	27.32~28.80	30.67±5.56	29.82~31.52	1.10±.12	1.08~1.12	0.51±1.34	0.30~0.71
*F*	317.663		404.395		202.386		72.769	
*P*^a^	<0.001		<0.001		<0.001		<0.001	

^a^:The Tukey-Kramer multiple comparison test was used to compare the DEM score indices for every two age groups. Vertical: age group 5>age group 6>age group 7>age group 8>age group 9>age group 10, age group 11, age group 12; Horizontal: age group 5>age group 6>age group 7>age group 8>age group 9, age group 10, age group 11, age group 12; Ratio: age group 5>age group 6>age group 7>age group 8, age group 9, age group 10, age group 11, age group 12; Errors: age group 5>age group 6>age group 7>age group 8, age group 9, age group 10, age group 11, age group 12.

### Derivation of DEM norms for Mandarin-speaking children

According to the DEM test manual, clinicians should compare a child’s test results with the appropriate norm tables and determine the percentile rank for each score. To construct the DEM norm tables for Cantonese-speaking children, the percentile ranks of each score in each age group were determined according to frequency distribution statistics. The norm tables (Table A, B, C, D, E, F, G and Table H in S1 File) for each age group in our study are shown in Supporting Information.

### Comparison of DEM scores for each age group with multinational norms

The current study is larger than the studies of the US and Cantonese (Hong Kong) populations and is similar in size to the study of Spanish-speaking individuals. Our study cohort and procedures are similar to those used to establish the US test norms. We compared our data with those of the DEM test authors [16], a study of DEM norms in Spanish-speaking children [20], and a study of DEM norms in a Cantonese-speaking population (Hong Kong) [18]. The mean values (standard deviation) are listed in Table 4. Most of the vertical and horizontal scores of the Mandarin-speaking children in all age groups were significantly different from those of the English-, Spanish- and Cantonese-speaking children in the other studies (independent *t*-test; the t1, t2, and t3 values for all age groups are shown in Table 4; *P1*, *P2 and P3*<0.001). The results indicated that the means of the vertical and horizontal scores in our study (for the Mandarin Chinese-speaking children) were significantly smaller than those of English-speaking children in all age groups (the t2 values for all age groups are shown in Table 4; *P2*<0.001 except for the vertical score in the 6-year-old group, which was *P2* = 0.081). In addition, the ratio score was not significantly different in the 8-year-old age group when compared with the English-speaking children (t2 = -1.52, *P2*>0.05, Table 4).

**Table 4:** Comparison of DEM scores Chinese (Mandarin) vs Chinese (Cantonese)/ English/ Spanish studies.

AgeGroup (years)		Chinese (Mandarin)	Chinese (Cantonese)	English	Spanish	*t*	*P*
	n	137	--	--	--		
5~	Vertical	88.31±25.91	--	--	--		
	Horizontal	140.86±58.87	--	--	--		
	Ratio	1.60±0.49	--	--	--		
	Error	12.50±10.92	--	--	--		
	n	238	53	52	115	t _1_; t_2_; t_3_	*P*_*1;*_ *P*_*2;*_ *P*_*3*_
6~	Vertical	62.84±17.97	50.98±13.16	63.11±15.59	86.30± 23.02	10.18*; -0.23; -20.14*	<0.001; 0.81;<0.001
	Horizontal	86.67±34.10	71.27± 18.45	98.26±32.61	146.90± 41.60	6.97*; -5.24*;-27.25*	<0.001; <0.001; <0.001
	Ratio	1.38±0.28	1.41±0.25	1.58±0.45	1.67±0.37	-1.90; -11.35*; -16.36*	0.059; <0.001; <0.001;
	Error	7.17±8.22	9.66±7.51	15.22±11.49	7.70±4.43	-4.67*;-15.11*; -0.99	<0.001; <0.001; 0.323
	n	183	63	75	115	t _1_; t_2_; t_3_	*P*_*1;*_ *P*_*2;*_ *P*_*3*_
7~	Vertical	50.48±9.72	43.34±8.82	54.83±9.20	58.27±13.04	7.73*; -19.57*; -7.84*	<0.001; <0.001; <0.001
	Horizontal	65.86±15.26	57.14±14.21	87.94±28.18	81.38± 26.91	9.93*; -6.06*; -29.91*	<0.001; <0.001; <0.001
	Ratio	1.31±0.22	1.32±0.16	1.60±0.91	1.70± 0.29	-0.47; -17.52*; -40.18*	0.641; <0.001; <0.001
	Error	4.26±6.19	5.59±6.72	12.50±12.91	6.97 ± 6.41	-2.90*; -17.99*; -3.50*	0.004; <0.001; <0.001
	n	195	54	93	240	t _1_; t_2_; t_3_	*P*_*1;*_ *P*_*2;*_ *P*_*3*_
8~	Vertical	43.64±9.14	38.50±7.99	46.76±7.89	49.60±9.65	7.85*; -4.78*; -9.12*	<0.001; <0.001; <0.001
	Horizontal	52.76±12.74	47.55±10.60	57.73±12.32	68.30±19.57	5.71*; -5.45*; -17.04*	<0.001; <0.001; <0.001
	Ratio	1.21±0.20	1.24±0.15	1.24±0.18	1.40±0.31	-1.52;-1.52; -12.43*;	0.130; 0.130; <0.001
	Error	1.86±3.18	3.96±3.74	4.61±6.91	5.55±5.97	-9.21*; -12.06*; -16.18*	<0.001; <0.001; <0.001
	n	156	50	84	238	t _1_; t_2_; t_3_	*P*_*1;*_ *P*_*2;*_ *P*_*3*_
9~	Vertical	37.18±7.76	36.53±6.61	42.33±8.20	42.37±7.46	1.04; -8.21*; -8.35*	0.298; <0.001; <0.001
	Horizontal	43.03±10.66	43.03±7.83	51.13±13.30	54.54±11.60	-0.003; -9.49*; -13.49*	0.997; <0.001; <0.001
	Ratio	1.16±.18	1.18±0.12	1.21±0.19	1.31±0.22	-1.26; -3.40*; -10.46*	0.201; 0.001; <0.001
	Error	1.45±3.83	2.98±3.28	2.17±4.10	1.69±2.66	-2.29*; -1.13; -0.47	0.010; 0.130; 0.320
	n	184	52	73	258	t _1_; t_2_; t_3_	*P*_*1;*_ *P*_*2;*_ *P*_*3*_
10~	Vertical	32.33±6.04	29.38±6.00	40.28±4.73	40.19±7.65	6.61*; -17.86*; -17.66*	<0.001; <0.001; <0.001
	Horizontal	36.04±6.98	33.69±8.72	47.64±10.11	47.24±10.37	4.58*; -22.54*; -21.77*	<0.001; <0.001; <0.001
	Ratio	1.12±.13	1.14±0.15	1.19±0.17	1.25±0.16	-1.81; -6.82*; -12.84*	0.071; <0.001; <0.001
	Error	0.71±1.48	1.56±2.16	1.91±2.68	0.81±1.76	-7.75*; -10.94*; -0.90	<0.001; <0.001; 0.372
	n	166	33	82	252	t _1_; t_2_; t_3_	*P*_*1;*_ *P*_*2;*_ *P*_*3*_
11~	Vertical	30.35±5.67	29.83±5.36	37.14±5.42	36.16±6.32	1.18; -15.43*; -13.20*	0.24; <0.001; <0.001
	Horizontal	32.83±6.48	32.87±7.03	42.62±7.61	43.49±10.04	-0.43; -39.21*; -41.48*	0.94; <0.001; <0.001
	Ratio	1.09±0.14	1.10±0.11	1.15±0.13	1.18±0.15	-0.36; -2.67*; -3.82*	0.36; 0.004; <0.001
	Error	0.55±1.51	1.70±2.72	1.68±2.34	0.51±1.68	-9.76*; -9.59*; 0.35	<0.001; <0.001; 0.725
	n	166	--	--	134	t _3_	*P*_*3*_
12~	Vertical	28.06±4.80	--	--	35.81±7.34	-11.00*	<0.001
	Horizontal	30.67±5.56	--	--	40.36±7.87	-12.47*	<0.001
	Ratio	1.10±0.12	--	--	1.12±0.12	-1.43	0.07
	Error	0.51±1.34	--	--	1.84±2.96	-5.17*	<0.001

*Statistically significant differences (*P<*0.001); Chinese (Mandarin) vs Chinese (Cantonese) (t _1,_*P*_*1*_); Chinese (Mandarin) vs English (t _2,_*P*_*2*_); Chinese (Mandarin) vs Spanish (t _3,_*P*_*3*_).

The total error scores were not significantly different for the 6- (t3 = 0.99, *P3* = 0.323, Table 4), 9- (t3 = 0.47, *P3* = 0.320, Table 4), 10- (t3 = -0.90, *P3* = 0.372, Table 4) and 11-year-old (t3 = 0.35, *P3* = 0.725, Table 4) age groups compared with the Spanish-speaking children. Significant differences were not observed for almost half of the test scores compared with the Cantonese-speaking children (the t1 and *P1* values are shown in Table 4), including the vertical-horizontal ratios for the 6-, 7- and 8-year-old groups, all of the test scores for the 9-year-old group (except the total error score), the vertical-horizontal ratio for the 10-year-old group, and the vertical and horizontal scores and the vertical-horizontal ratio for the 11-year-old group. The remaining scores were statistically significant (*P1*<0.001, Table 4).

### Reliability and validity of the DEM test

Evaluating the reliability and validity of the Mandarin Chinese DEM scores provides an effective means of evaluating the use of the DEM test in the Chinese population. In the current study, a retest was conducted to assess the reliability of this test in Nanjing, China. The time reliability test and tester reliability test were conducted separately. To evaluate time reliability, we re-measured a random sample of 180 children during a two-week period. The means and standard deviations of the DEM scores were similar for the two tests (Table 5). The two tests performed at different times showed correlation coefficients of 0.84, 0.90, 0.92 and 0.91 for the vertical score, horizontal score, ratio score and total error score, respectively, indicating that the tests had high time reliability. To assess tester reliability, the two testers were switched, and they then re-measured a random sample of 180 children. The correlation coefficients of the two tests performed by each tester were 0.99 for the vertical score, 0.99 for the horizontal score, 0.99 for the vertical-horizontal ratio, and 0.99 for the total error score (Table 6). These results indicated the good reliability of the DEM test. Significant declining trends were observed for the vertical score, horizontal score and total error score with increasing age (Table 3). The internal consistency validity was investigated by comparing the correlation coefficients among the three eye movement indices, which were significantly different (the vertical score was *P*<0.05 and the horizontal score and ratio score were *P*<0.001, Table 7). Our results indicate that the DEM test norms for the Mandarin Chinese-speaking children can be used to accurately assess their clinical visual-verbal ocular motor functions.

**Table 5:** Time reliability of the DEM test for Chinese (Mandarin)-speaking Chinese children.

n = 180	First time test	Second time test	*r*	*p*
Vertical	49.13±12.24	45.88±8.16	0.84	0.489
Horizontal	58.44±17.74	55.56±14.27	0.90	0.104
Ratio	25.91±6.25	26.05±5.58	0.92	0.175
Error	40.73±11.19	39.53±6.95	0.91	0.399

**Table 6:** Tester reliability of the DEM test for Chinese (Mandarin)-speaking Chinese children.

n = 180	First time test	Second time test	*r*	*P*
Vertical	37.69±11.12	37.72±11.37	0.99	0.489
Horizontal	44.33±16.48	44.89±17.08	0.99	0.104
Ratio	1.16±0.18	1.17±0.19	0.99	0.175
Error	1.57±3.73	1.84±4.98	0.99	0.399

**Table 7:** Correlation analysis of the eye movement index of the DEM.

eye movement index	Vertical	Error	Ratio
Horizontal	0.772**	0.580**	0.710**
Vertical	--	0.428**	0.138*
Error	--	--	0.411**

*Statistically significant differences (*P<*0.05),

**Statistically significant differences (*P*<0.001).

## Discussion

Children with poor reading skills include those with dyslexia, non-dyslexic reading disabilities and poor linguistic cognition and non-linguistic perceptual cognitive processing, as well as other problems. Appropriate norms for specific languages should be used to allow examiners to determine accurate DEM scores and to provide proper diagnoses for reading problems.

The DEM test is a standardized clinical test that is recommended for the assessment of children with reading problems and other learning-related vision problems [13, 21]. In the present study, we used stratified random sampling to recruit 1,425 children aged 5 to 12 years from eight kindergartens and eight primary schools in Nanjing. Similarly to the original DEM study [17], our participants were recruited from urban public and suburban public schools. Hence, the present study achieved sample representativeness. Importantly, our study assessed preschool children aged 5 to 6 years, whereas other studies have not evaluated this age group. Therefore, our DEM test can aid in the early identification of children at risk of reading problems other than dyslexia, thus providing a more economical and beneficial alternative to later intervention and treatment.

Mandarin Chinese has characteristics similar to Cantonese Chinese. For example, both are ideographic languages that differ from phonetic languages, such as English. Thus, we proposed the use of “adjusted vertical scores” for the vertical score norms for the Mandarin Chinese-speaking children in view of the relatively high incidence of vertical errors in our study. Our results showed significant declining trends in the vertical and horizontal scores and in the total error score with increasing age and grade level. Our results are consistent with the general notion that a child should be able to read faster with age because of the gradual development and maturity of automaticity and eye movement. No significant differences were observed in the horizontal score and the vertical score among the 10-, 11- and 12-year-old age groups. Moreover, no significant differences in the ratio score and the total error score were observed among the 8-, 9-, 10-, 11- and 12-year-old age groups. This finding (the change in horizontal and vertical scores) is in accordance with the notion that a child’s reading speed will plateau as he/she ages. Furthermore, our test involved the use of simple single-digit items; thus, it was rather easy for the adolescents to perform.

Our results also showed that the Mandarin Chinese-speaking children were able to complete the vertical and horizontal DEM tests significantly more rapidly than both the English- and Spanish-speaking children in all age groups. This result suggests that the eye movement speed of the Chinese children with regard to the movement indices was significantly faster than those of their English and Spanish counterparts. The Chinese children obtained significantly lower vertical-horizontal ratio and total error scores than the English and Spanish children did. This finding may be the result of several factors. First, Chinese is an ideographic language, and numbers are easier to pronounce in Chinese than in English [22]. Therefore, the duration of each eye movement index differed between the two populations. However, the results of the DEM test are similar between phonetic languages, such as English and Spanish, as demonstrated by previous studies of Spanish and English children performed by Fernandez-Velazquez and Fernandez-Fidalgo [20] and Jiménez et al. [23]. However, differences among phonetic languages were found in Baptista et al.’s study [24], and they considered that different languages, educational systems and cultures may explain these differences. Moreover, we cannot determine exactly what factor(s) might account for the DEM scores differences at this time. Second, the time at which reading training is initiated differs between Western countries and China. In China, children often begin reading training earlier than their American counterparts [25]. Chinese parents notice the academic development of their children during kindergarten [25]. The vertical and horizontal scores of the children in our study differed significantly from those of the Cantonese-speaking children in the 6-, 7-, 8-, and 10-year-old age groups, which might be related to educational and economic level differences between the two samples. Jiménez et al. [23] found no differences in the DEM test results between Spanish-speaking children and the original group of English-speaking children described by Garzia and colleagues [18]; therefore, it is possible that differences in language, cultural background, and educational systems affect DEM scores.

In the current study, we excluded subjects with neurological disease, physical disabilities and mental retardation and those who could not complete the DEM test because of an inability to stay on task even after redirection. The experienced examiners were able to detect reading errors easily [26]. The manner in which the DEM test is administered might also contribute to its accuracy. The DEM test-retest has been shown to have excellent reliability when it is used to evaluate patients undergoing vision therapy evaluations [27]. The present study indicated good test-retest reliability of the DEM. The internal consistency was investigated by comparing the correlation coefficients among the three eye movement indices. Only DEM tests with sufficient reliability will yield accurate measurements.

In addition, the time reliability test and tester reliability test were conducted separately. For the time reliability test, the correlation coefficient of the two tests performed at different times showed a vertical score of 0.84, a horizontal score of 0.90, a vertical-horizontal ratio of 0.92, and a total error score of 0.91. Accurate assessments of reading errors were performed by two experienced examiners. To assess tester reliability, we switched the two testers, who then re-measured a random sample of 180 children. The correlation coefficients of the two sets of tests were 0.99 for the vertical score, 0.99 for the horizontal score, 0.99 for the vertical-horizontal ratio, and 0.99 for the total error score. These results indicated the high reliability of our localized DEM test. Our results are consistent with those of Garzia et al. [16] and Jimenez et al. [23], who reported reference values that were relatively similar to those of the US population, with the exception of the 5-year-old age group in the Spanish study.

Our analysis of the DEM test allowed us to establish DEM test norms for children in Nanjing (aged 5 to 12 years). Because in China, children often begin reading training earlier than their American counterparts [22], Chinese parents emphasize academic development at an early age. By the time children enter kindergarten in China, they have already learned how to read Arabic numerals. Second, Nanjing is a moderately developed city. The city participates in scientific research, including research related to childhood obesity in China. Third, the subjects who participated in this study had already passed a DEM pretest. Moreover, all of the participants were recruited from the main urban and suburban areas of Nanjing; thus, the selected participants accurately reflect the entire Chinese population. Thus, the norms of the DEM test will be proposed for and used to evaluate Mandarin Chinese-speaking children throughout China. A considerable number of statistically significant differences between the Mandarin Chinese population study group and the other groups were observed. Finally, the DEM test norms for Mandarin Chinese-speaking Chinese children showed good reliability and validity and could be used to assess their clinical visual-verbal ocular motor functions.

## Supporting Information

S1 File The DEM norms table for Cantonese-speaking children. The DEM norms table for Cantonese-speaking children from age 5 years to age 5 years, 11 months (Table A); The DEM norms table for Cantonese-speaking children from age 6 years to age 6 years, 11 months (Table B); The DEM norms table for Cantonese-speaking children from age 7 years to age 7 years, 11 months (Table C); The DEM norms table for Cantonese-speaking children from age 8 years to age 8 years, 11 months (Table D); The DEM norms table for Cantonese-speaking children from age 9 years to age 9 years, 11 months (Table E); The DEM norms table for Cantonese-speaking children from age 10 years to age 10 years, 11 months (Table F); The DEM norms table for Cantonese-speaking children from age 11 years to age 11 years, 11 months (Table G); The DEM norms table for Cantonese-speaking children from age 12 years to age 12 years, 11 months (Table H).
(DOC)
