# Correction: Developmental Eye Movement (DEM) Test Norms for Mandarin Chinese-Speaking Chinese Children

**DOI:** 10.1371/journal.pone.0154909

**Published:** 2016-04-28

**Authors:** The PLOS ONE Staff

The eighth and ninth authors, Qin Hong and Xia Chi, should be noted as corresponding authors. The contact email for Qin Hong is rambler_hq@163.com. The publisher apologizes for the error.
