# Supplementary material for: Developmental Eye Movement (DEM) Test Norms for Mandarin Chinese-Speaking Chinese Children
Source: PLoS One. 2016 Feb 16;11(2):e0148481. doi: 10.1371/journal.pone.0148481 (PMC4755595; doi:10.1371/journal.pone.0148481)
Supplement: S1 File — The DEM norms table for Cantonese-speaking children from age 5 years to age 5 years, 11 months (Table A); The DEM norms table for Cantonese-speaking children from age 6 years to age 6 years, 11 months (Table B); The DEM norms table for Cantonese-speaking children from age 7 years to age 7 years, 11 months (Table C); The DEM norms table for Cantonese-speaking children from age 8 years to age 8 years, 11 months (Table D); The DEM norms table for Cantonese-speaking children from age 9 years to age 9 years, 11 months (Table E); The DEM norms table for Cantonese-speaking children from age 10 years to age 10 years, 11 months (Table F); The DEM norms table for Cantonese-speaking children from age 11 years to age 11 years, 11 months (Table G); The DEM norms table for Cantonese-speaking children from age 12 years to age 12 years, 11 months (Table H). (DOC) [file pone.0148481.s001.doc]

**Table A. The DEM norms table for Cantonese-speaking children from age 5 years to age 5 years, 11 months**

| Percentile | Vertical | Horizontal | Ratio | **Error** |
| --- | --- | --- | --- | --- |
| 95 | 60.05 | 82.01 | 0.97 | 0.00 |
| 85 | 65.15 | 87.55 | 1.18 | 5.00 |
| 75 | 71.50 | 98.95 | 1.28 | 5.00 |
| 50 | 91.00 | 132.17 | 1.49 | 10.00 |
| 25 | 116.75 | 182.01 | 1.68 | 20.00 |
| 15 | 126.85 | 205.87 | 1.89 | 26.70 |
| 5 | 135.00 | 301.97 | 3.12 | 36.90 |

**Table B. The DEM norms table for Cantonese-speaking children from age 6 years to age 6 years, 11 months**

| Percentile | Vertical | Horizontal | Ratio | Error |
| --- | --- | --- | --- | --- |
| 95 | 47.60 | 64.90 | 1.03 | 0.00 |
| 85 | 59.00 | 81.70 | 1.27 | 0.70 |
| 75 | 66.50 | 97.14 | 1.31 | 3.50 |
| 50 | 79.00 | 121.95 | 1.49 | 10.00 |
| 25 | 98.00 | 165.19 | 1.78 | 19.00 |
| 15 | 117.20 | 186.60 | 2.00 | 22.00 |
| 5 | 126.70 | 259.86 | 2.66 | 32.10 |

**Table C.** **The DEM norms table for Cantonese-speaking children from age 7 years to age 7 years, 11 months**

| Percentile | Vertical | Horizontal | Ratio | Error |
| --- | --- | --- | --- | --- |
| 95 | 38 | 46 | 1.01 | 0 |
| 85 | 42 | 51 | 1.10 | 0 |
| 75 | 43 | 55 | 1.17 | 0 |
| 50 | 48 | 63 | 1.30 | 2 |
| 25 | 56 | 75 | 1.41 | 5 |
| 15 | 59 | 82 | 1.51 | 9 |
| 5 | 69 | 95 | 1.70 | 16 |

**Table D. The DEM norms table for Cantonese-speaking children from age 8 years to age 8 years, 11 months**

| Percentile | Vertical | Horizontal | Ratio | Error |
| --- | --- | --- | --- | --- |
| 95 | 31 | 36 | 0.97 | 0 |
| 85 | 35 | 40 | 1.04 | 0 |
| 75 | 37 | 44 | 1.09 | 0 |
| 50 | 42 | 50 | 1.19 | 0 |
| 25 | 49 | 59 | 1.32 | 2 |
| 15 | 53 | 67 | 1.38 | 5 |
| 5 | 60 | 77 | 1.52 | 10 |

**Table E. The DEM norms table for Cantonese-speaking children from age 9 years to age 9 years, 11 months**

| Percentile | Vertical | Horizontal | Ratio | Error |
| --- | --- | --- | --- | --- |
| 95 | 27 | 31 | 0.94 | 0 |
| 85 | 30 | 34 | 1.04 | 0 |
| 75 | 32 | 36 | 1.07 | 0 |
| 50 | 36 | 41 | 1.15 | 0 |
| 25 | 43 | 49 | 1.24 | 1 |
| 15 | 46 | 53 | 1.31 | 2 |
| 5 | 51 | 60 | 1.44 | 7 |

**Table F. The DEM norms table for Cantonese-speaking children from age 10 years to age 10 years, 11 months**

| Percentile | Vertical | Horizontal | Ratio | Error |
| --- | --- | --- | --- | --- |
| 95 | 24 | 26 | 0.91 | 0 |
| 85 | 26 | 29 | 1.00 | 0 |
| 75 | 28 | 31 | 1.03 | 0 |
| 50 | 31 | 35 | 1.12 | 0 |
| 25 | 36 | 40 | 1.20 | 1 |
| 15 | 39 | 44 | 1.25 | 1 |
| 5 | 44 | 48 | 1.35 | 5 |

**Table G. The DEM norms table for Cantonese-speaking children from age 11 years to age 11 years, 11 months**

| Percentile | Vertical | Horizontal | Ratio | Error |
| --- | --- | --- | --- | --- |
| 95 | 21 | 23 | 0.90 | 0 |
| 85 | 24 | 27 | 0.97 | 0 |
| 75 | 26 | 29 | 1.00 | 0 |
| 50 | 30 | 32 | 1.10 | 0 |
| 25 | 34 | 37 | 1.18 | 1 |
| 15 | 36 | 40 | 1.21 | 2 |
| 5 | 41 | 43 | 1.28 | 3 |

**Table H. The DEM norms table for Cantonese-speaking children from age 12 years to age 12 years, 11 months**

| Percentile | Vertical | Horizontal | Ratio | Error |
| --- | --- | --- | --- | --- |
| 95 | 21 | 22 | 0.90 | 0 |
| 85 | 23 | 25 | 0.97 | 0 |
| 75 | 25 | 27 | 1.03 | 0 |
| 50 | 27 | 30 | 1.09 | 0 |
| 25 | 31 | 34 | 1.17 | 0 |
| 15 | 33 | 37 | 1.21 | 1 |
| 5 | 37 | 41 | 1.32 | 4 |
